# Global and national actions to prevent trade in substandard and adulterated medicines

**DOI:** 10.1371/journal.pgph.0004024

**Published:** 2025-02-20

**Authors:** Richard Wilder, Sam Halabi, Lawrence O. Gostin

**Affiliations:** 1 University of New Hampshire, Franklin Pierce School of Law, Concord, New Hampshire, United States of America; 2 O’Neill Institute for National and Global Health Law, Georgetown University Law Center, Washington, DC, United States of America; 3 Department of Health Management and Policy, School of Health, Georgetown University Medical Center, Washington, DC, United States of America; Federal University Birnin Kebbi, NIGERIA

## Abstract

Recent mass-poisoning events caused by substandard medicines placed in global markets raise the question as to what more can be done to stop it. Efforts have been underway for years at the World Health Organization and other multilateral fora, such as the National Academies of Medicine in the U.S. While these efforts have led to improvements, several gaps remain which, unless addressed, will allow this problem in international trade to continue. Some countries have failed to implement the standards set by the WHO by not fully empowering national regulatory agencies to lead national efforts. Further, some countries do not fully exercise their responsibility under WHO standards to confirm that the information in documents accompanying internationally traded medicines is complete, accurate, and current. It is time to revisit international standards in this area to determine the causes of these problems and to address them.

## Introduction

In late 2022, the world was shocked by mass poisoning events in The Gambia, Uzbekistan, and Indonesia, where more than 300 children – mostly aged under 5 – died of acute kidney injury associated with contaminated cough syrups that were manufactured in India. Some reports stated that suspected substandard medicinal syrups were made in Indonesia. The medicines, over-the-counter cough syrups, had high levels of diethylene glycol and ethylene glycol. The World Health Organization (WHO) said that “[t]hese contaminants are toxic chemicals used as industrial solvents and antifreeze agents that can be fatal even taken in small amounts and should never be found in medicines.” [1]. As well as the three countries above, the WHO reports that the Philippines, Timor L’este, Senegal, and Cambodia may potentially be impacted because they may have had the medicines on sale [2]. The WHO called for action across its 194 member states to prevent more deaths.

However, the import and use of substandard products is not limited to low and middle-income countries (LMICs). In early 2023 the US Centers for Disease Control (USCDC) and Food and Drug Administration (FDA) issued warnings against the purchase or use of eyedrops made in India [3]. The eye drops had led to a multistate outbreak of an extensively drug-resistant strain of *Pseudomonas aeruginosa* that, when used, had led to eye infections, loss of sight, and death.

There is also a history of patients in the U.S. suffering from the use of diethylene glycol in medicines. In the 1930’s, sulfanilamide had shown promise in the United States when used in tablet and powder form to treat streptococcal infections. A manufacturer decided to produce a liquid formulation to expand the market- particularly for children. In 1937, such a formulation was created by dissolving sulfanilamide in a solvent, diethylene glycol, untested for safety and unaware of its deadly effect when consumed. This product was manufactured and shipped widely and quickly led to the deaths of more than 100 people, many of them children. Drug regulation in the US at that time was known to be inadequate. There were no effective legal tools to prevent the introduction of unsafe medicines into the US market. The FDA only charged the manufacturer with “misbranding” because the syrup was branded as an “Elixir” but did not include alcohol, which such branding required at the time. The incident accelerated the final enactment in 1938 of the Federal Food, Drug, and Cosmetic Act, the basis for the FDA’s current regulation of medicines.

These incidents focused the attention of national drug authorities and international organizations, including the WHO, on ways to remedy the problem. These efforts did lead to positive steps, including an approach developed under the auspices of the WHO to put more information and data in the hands of countries to assess the safety of imported drugs. The problem persists, however, and more work needs to be done at the international and national levels to improve both the current systems and the way in which they are used.

## Efforts by National Academies of Sciences, Engineering and Medicine (NASEM) and World Health Organization (WHO) to address the problem of substandard medicines in international trade

In 2013, the Institute of Medicine of the National Academies of Sciences, Engineering and Medicine (now the National Academy of Medicine) published a report by its Committee on Understanding the Global Public Health Implications of Substandard, Falsified, and Counterfeit Medical Products [4]. That report chronicled mass poisoning events in Nigeria and Panama, where diethylene glycol was fraudulently sold as pharmaceutical-grade glycerin and used in cough syrups, leading to hundreds of deaths. The report issued recommendations and observations – many of which have been taken up by the World Health Organization, as discussed below. Four of the observations made in the IOM Committee report continue to vex efforts to prevent substandard products from entering international trade:

- The illegal trade and manufacture of medicines are a global problem, disproportionately affecting low- and middle-income countries.
- When regulatory checks on production are inconsistent, procurement practices can help ensure that honest manufacturers get the largest market share.
- In countries where state and federal governments share regulatory oversight, the division of responsibility is not always clear.
- A reliable system for tracking and tracing drugs through the distribution chain would reduce the likelihood of illegitimate medicines reaching patients.

The World Health Assembly adopted a Resolution in 2012 to establish a Member State Mechanism to address substandard and falsified medical products [5]. The resolution passed, reflecting increased global concerns about such products and the health and socioeconomic harms they cause.

## WHO Global Surveillance and Monitoring System for substandard and falsified medical products

In 2013, WHO launched the Global Surveillance and Monitoring System to encourage countries to report incidents of substandard and falsified medical products in a structured and systematic format, to help develop a more accurate and validated assessment of the problem [6]. The role of the WHO as the secretariat for that System includes:

- providing technical support in emergencies, links incidents between countries and regions, and issues WHO medical product alerts;
- gathering a validated body of evidence to more accurately demonstrate the scope, scale and harm caused by substandard and falsified medical products and identify the vulnerabilities, weaknesses and trends;
- publishing medical product rapid alerts in cases where there is a serious risk to public health affecting a wide geographic area; and
- strengthening National and Regional Medicines Regulatory Authorities in preventing, detecting and responding to SF medical products.

In parallel, the World Health Assembly “established the Member State Mechanism to address the issue of tackling substandard and falsified medical products... ” [7]. Notwithstanding the significant efforts to address the problem of international trade in contaminated medicines, that trade continues. The WHO issued a medical product alert regarding five different syrup and suspension medicines made by a company in Pakistan contaminated with diethylene glycol and ethylene glycol. These substandard medicines were detected in the Maldives, Pakistan, Belize, Fiji and Lao People’s Democratic Republic [8]. The problem was not detected and addressed at the point of manufacture but at the point of entry into the Maldives and using a new thin-layer chromatography test to identify these poisons. This medical product alert again pointed out the failure to detect such problems with substandard products at the point of manufacture or export. The WHO recognized this same problem with substandard liquid dosage medicines contaminated with diethylene glycol and/or ethylene glycol and shipped to at least Uzbekistan and Cambodia [9]. There have been similar medical product alerts issued by the WHO from 2022 to the present for medicinal syrups including diethylene glycol and/or ethylene glycol and imported by various LMICs [10].

Manufacturers and national drug regulatory agencies must also closely monitor the supply chain of ingredients for finished medicinal products. A recent medical product alert from the WHO [11] revealed that the Drug Regulatory Authority of Pakistan found oral liquid medicines potentially contaminated with ethylene glycol. The source of the contamination was identified at the manufacturer as drums falsely labeled as containing propylene glycol (an excipient and solvent regarded as safe for use in medicines). The drums contained ethylene glycol - an ingredient poisonous to humans. This highlights the need for manufacturers to follow GMP standards for excipients used in pharmaceutical products and for manufacturers and national regulatory authorities to be diligent in maintaining this standard.

Solutions to problems with manufacturing and distributing substandard medical products fall into three categories: prevention, detection, and response [12]. Solutions directed at the “prevention” of trade in such products are the most effective as they address the problem at its origin. Certainly, detection and response solutions to mitigate trade in substandard products are critical and need to be vigorously implemented. But detection – such as the use of the thin layer chromatography test by the Maldives – can be too costly for many LMICs, and response tools come into play only when the problem has occurred, and substandard products are being distributed and used. Ideally, the solution to the problem of substandard medicines is to prevent them being made or shipped in the first instance. However, systems intended to prevent the manufacture and global distribution of substandard medicinal products are not working as intended. For example, the aggregate observed failure rate of tested samples of substandard and falsified medicines in low- and middle-income countries is approximately 10.5% [13]. This number varies from country to country since, as discussed further below, some LMICs can better test and find substandard products at the point of import and stop them from entering the local market. Other countries do not have the necessary resources. They are therefore more reliant on statements by the manufacturing companies and their national regulatory agencies that the products are safe both as designed and as manufactured.

The system for the issuance of Certificates of Pharmaceutical Product (the “CoPP System”) was developed by the WHO and its Member States and has been an essential addition to the “prevention” tool kit. It was established to help reduce the introduction of substandard products to the market. This is in keeping with the requirement that pharmaceutical manufacturers possess a “comprehensive system of quality assurance must be founded on a reliable system of licensing and independent analysis of the finished product, as well as upon assurance obtained through independent inspection that all manufacturing operations are carried out in conformity with accepted norms, referred to as [Good Manufacturing Practices].” [14]. Certainly, if such data were reliable and confirmed independently upon the importation of medicines, it would be much less likely that substandard medicines would be made and introduced into the market. That is not happening, however.

If a Member State wishes to use the CoPP System to support the export of pharmaceutical products, it must possess the following:

- an effective national licensing system, not only for pharmaceutical products but also for responsible manufacturers and distributors;
- GMP requirements, consonant with those recommended by WHO, to which all manufacturers of finished pharmaceutical products are required to conform;
- effective controls to monitor the quality of pharmaceutical products registered or manufactured within its country, including access to an independent quality control laboratory;
- a national pharmaceuticals inspectorate, operating as an arm of the national drug regulatory authority, and having the technical competence, experience, and resources to assess whether GMP and other controls are being effectively implemented and the legal power to conduct appropriate investigations to ensure that manufacturers conform to these requirements by, for example, examining premises and records and taking samples;
- administrative capacity to issue the required certificates, institute inquiries in the case of a complaint, and to expeditiously notify both WHO and the competent authority in any Member State known to have imported a specific product that is subsequently associated with a potentially serious quality defect or another hazard [15].

One weakness in the CoPP System is that the Member States make their own determinations that their laws, regulations, and resources meet the foregoing requirements. Generally, one should be wary of any system that relies entirely on self-assessment to ensure the data and conclusions are accurate and complete [16]. Given the vital public health and commercial interests at stake, this general rule should apply more forcefully to the CoPP System. When discussing the eligibility for participation in the CoPP System, the WHO emphasizes reliance on self-assessment, stating that the System contains “no provision, under any circumstance, for external inspection or assessment, either of a competent national authority or of a manufacturing facility.” India still needs to fully implement the CoPP System to achieve its stated goals, a failure that has more to do with the self-assessment approach to compliance than a lack of resources or capabilities to achieve full compliance. This failure has implications for both India and the world given India’s stated aspiration to be the “Pharmacy of the World.” [17].

The shortcomings of a self-assessment system can be overcome through transparency (providing the data or information upon which the self-assessment is based to allow independent confirmation) and oversight (in particular of the national regulatory authorities of manufacturing countries). That oversight should allow reviews by importing countries and other interested parties to independently substantiate the conclusions reported in the CoPP.

## Tracking and tracing medicines in international trade

We agree with the IOM Study’s observation that a “reliable system for tracking and tracing drugs through the distribution chain would reduce the likelihood of illegitimate medicines reaching patients.” The Traceability and Verification (or “TRVST”) System, developed and launched under the auspices of UNICEF [18], is a viable system for this purpose. The TRVST System includes a global repository that stores product master data, serial numbers, market authorization data, and product pack data. Manufacturers upload this data into the TRVST System. Users of the System—such as pharmacists, front-line healthcare workers, regulatory authorities, and customs agents—may then use a phone-based application to scan product barcodes to verify their authenticity in real-time. Any verification failure or suspect activities will trigger an alert from the TRVST System to the appropriate manufacturers and regulatory authorities [19].

The TRVST System should be considered a valuable tool for identifying and removing products from the market that are not authenticated as coming from a legitimate, licensed manufacturer. Regulatory authorities may also use it to track down products from a particular manufacturer reported as substandard, defective, or potentially dangerous.

## Country experiences with imported substandard medicines

The experience of three countries in addressing the risk of importation of substandard medicines illustrate:

(i) an “all of government” approach (the Gambia),
(ii) the experience of a country (Nigeria) in assessing the accuracy and completeness of CoPPs that accompany imported medicines, and
(iii) steps an importing country can take to deal with repeat offenders – whether manufacturers or exporting countries that allow the manufacture and export of substandard medicines (Sri Lanka).

### The Gambia

When it became apparent that substandard medicines had been imported into The Gambia, leading to injuries and death, a Parliamentary Inquiry was quickly launched under the Select Committee on Health, Disaster, Refugees and Humanitarian Relief [20]. The findings of that Select Committee were included in a report issued on December 20, 2022. The report is critical to understanding the regulatory failures that occurred in India – the source of the substandard medicines imported by The Gambia – which led to the public health disaster there.

The Gambian Select Committee confirmed that medicinal syrups contaminated with diethylene glycol and ethylene glycol had been imported into their country and that the source of those products was India. The Select Committee was informed that the imported medicinal syrups were inspected upon arrival “and everything was satisfactory because all the products came with the required certificate of analysis from the Manufacturer and all the certificates indicating that the products are of good quality and fit for use.”

The certificates were found to be in order. Though not stated in the report of the Parliamentary Inquiry, those certificates likely included a CoPP. They were likely found in order if they properly identified the manufacturer of the medicine and that the CoPP was issued by a drug authority in India. The Gambian authorities, however, had to accept on faith the statements made in the CoPP about the safety of the medicine. The reality was that the products were not of good quality nor fit for use. Reliance on the Indian regulatory authorities and the Indian companies having made the medicines to have conducted the required inspections of manufacturing facilities and analysis of the medicine was not well founded. A lack of confidence in the content of a CoPP from a given manufacturer or country defeats the purpose of issuing one in the first place.

### Nigeria

Nigeria’s National Agency for Food and Drug Administration and Control (NAFDAC) conducts pre-shipment testing of drugs intended to be shipped to Nigeria from China and India [21]. NAFDAC provided 84 CoPPs for this study [22]. The 84 CoPPs are in the possession of the authors. All of those CoPPs correspond to drugs intended for shipment from India to Nigeria. All of those 84 drugs were tested through the Clean Report of Inspection and Analysis (CRIA) scheme, which provides for local, pre-shipment testing and control for medicines made in India and China. NAFDAC reported that all 84 drugs failed testing and were stopped from entering the Nigerian market.

All of the CoPPs were issued by State Drug Authorities rather than the national authority in India (the Central Drugs Standard Control Organization (CDSCO)) as required by the CoPP System [23] and Indian national law [24]. The 84 CoPPs that were reviewed showed inconsistencies between various State Drug Authorities in the way these CoPP certificates are granted. Some states require the manufacturer to provide a “summary basis” for issuing the CoPP, others don’t. Answers to questions 2A.3–4 and 2A.3–5 in the CoPP form provided by the WHO [25] vary significantly from state to state.

More troubling, however, is the failure to include key information or data in the CoPPs showing that the drug and its manufacturing facility satisfies the requirements specified in the model CoPP. Of the 84 CoPPs that were studied, none included any clinical or safety information or data (or links thereto) to support the conclusions stated in the CoPPs. The factual basis upon which these State Drug Regulatory authorities issued these CoPP certificates needs to be available to importing country authorities. The response to questions about GMP compliance appears to be checked off mechanically without evidence as to how the conclusion was reached. All the CoPPs indicate that the State Drug Regulators inspected the manufacturing facility that manufactured the drug for which the CoPP is being requested. But the CoPPs did not provide any evidence from such inspections – such as findings or inspection reports from the drug regulators in the country of manufacture or, if deficiencies are found, the corrective and preventive actions recommended and/or taken by the manufacturer. Further, most of the 84 CoPP certificates indicate that the medicinal product for export is registered and sold in the country of origin, but it has not been possible to substantiate those claims across many of the CoPPs examined. This lack of transparency undercuts the utility of the CoPP System as the expectation is that importing countries will rely on the CoPP as proof that safety, efficacy and manufacturing standards have been met. Put another way, the 84 CoPP certificates reviewed would have conveyed the same information if they contained only one checkbox stating (falsely) that “all applicable standards have been met.”

Another case study in Nigeria involves Telmisartan [26], a drug for the treatment of high blood pressure (hypertension) It is an essential drug for many people with heart disease as it can reduce the risk of stroke, heart attack, or other heart problems. The drug was manufactured by an Indian generic manufacturer (Micro Labs Ltd. Located in the State of Sikkim, India) and imported into Nigeria and a CoPP was included at the time of import along with a Certificate of Analysis from India showing 92% Active Pharmaceutical Ingredient (“API”) by assay (HPLC). Based on the documents alone, this appears acceptable as the monograph for Telmisartan published by the U.S. Pharmacopeia requires that “Telmisartan Tablets contain NLT 90.0% and NMT 110.0% of the labeled amount of telmisartan (C33H30N4O2).” [27]. However, when the drug was tested by NAFDAC after import into Nigeria, it was found to contain only 78% of the API – clearly out of specification according to the USP monograph. Moreover, there was at least one known patient on this drug that suffered an adverse event (“AE”) after taking the substandard drug and the AE was causally linked to the substandard drug.

It is clear, then that the CoPPs provided by authorities in India lack sufficient data or information that would enable corroboration of the accuracy of the CoPPs being approved. The fact that the information and data contained in the CoPPs were found to be incorrect through testing under the CRIA scheme further throws into question the reliability of CoPPs issued in India. Whether this is a consequence of the CoPPs being issued by state drug regulatory authorities in India (which is in contravention of WHO CoPP guidelines and India’s national policy) rather than the national Central Drugs Standard Control Organization should be further explored by the WHO.

### Sri Lanka

A recent study [28] investigated medicines withheld and recalled from the market in Sri Lanka to identify the types of defects, their total numbers, therapeutic categories, pharmaceutical dosage forms, and country of manufacturer during the study period. The authors of the study found that some manufacturers in India were “accountable for repetitive withholdings and recalls, which reflects the ignorance of quality control measures and weak regulatory inspections as a violation of Good Manufacturing Practice (GMP).” The authors expanded on the latter point saying, “these findings demonstrate the need for a control capacity in regulatory agencies and legislation that can impose relevant sanctions when necessary.”

## Conclusions and next steps

The CoPP System, as currently used for both manufacturing and exporting countries, is not adequate to reliably perform its stated objectives due to missing or inaccurate information. There are measures, however, that can be taken to improve the desired results for the CoPP System.

The information and data included in a CoPP are provided by the company manufacturing the medicine and that company’s national regulatory authority. Therefore, the accuracy and completeness of that data and information is entirely up to them. Any system that ensures the accuracy and completeness of a process of this sort must be transparent and allow for oversight of that system by the countries that use it to ensure that the desired degree of accuracy and completeness of the information is achieved. Data or information to confirm or support conclusions indicated on the CoPP can be more easily provided to countries or companies of import if an electronic CoPP is used with such data or information included via attachments or hyperlinks [29].

One step would be to *require* greater visibility of the information or data supporting CoPP statements. For example, information about inspections of facilities to determine if they meet GMP requirements would include when inspections were conducted, by whom, whether there were restrictions as to facilities or product production lines that were inspected, and what was observed during the inspections and if any corrections were required to be made (and then completed by the manufacturer). And, if there were corrections required in manufacturing, when and how they were completed and further inspected. Further, any data about the product itself should be available to the importing country– in particular analysis of ingredients for the medicine (both active ingredients and excipients) and post-manufacturing analyses of the medicine to ensure compliance with product specifications. All the foregoing information would be easier to provide and to review if it were made available in a standard electronic format. The purpose of requiring this information is to have information to guide any additional studies that a given importing country may need.

An additional step could be taken by the country of import as done by NAFDAC in Nigeria through its CRIA System which provides qualified inspectors, acting on behalf of NAFDAC, that inspect manufacturing facilities in India and China. This step may be performed as a backup or confirmation to the analyses done by the manufacturer and the national regulatory authority in the country of export and reported in the CoPP. And the overall expense of conducting such analyses of imported medicines could be reduced if directed by a risk-adjusted approach based on the additional information or data outlined above. Indeed, the additional information or data may reduce concerns about the CoPP System overall or that system as applied by certain countries to warrant continued reliance on it.

In addition to improved visibility to the data and information behind the statements made in a CoPP, an independent review of how the CoPP System is operated at the national level should be undertaken. Recall that the CoPP System “contains no provision, under any circumstance, for external inspection or assessment, either of a competent national authority or of a manufacturing facility.” That said, there is nothing in the CoPP System that would prevent a WHO Member State/importer from taking direct action to secure necessary changes to the operation of the CoPP System by manufacturers and the National Regulatory Authorities in countries that export to them. That is, a Member State/importer may require, as a condition of the export of medicines to their country, that the Member State/exporter must increase transparency as to the factual basis for the issuance of a CoPP. Such increased transparency should apply to: (i) the data and information that formed the basis for the issuance of a CoPP, (ii) the steps or process for making the decision to issue a CoPP, and(iii) the process whereby the manufacturer and/or issuing authority of the CoPP may correct mistakes or shortcomings to the CoPP if discovered after shipment of the medicines. That last point on transparency could be improved if a Member State/importer could require such additional information and data if later product testing shows that the issuance of the original CoPP was not well founded.

A further step could be action taken by National Regulatory Authorities that are members of regional bodies – such as the African Union for its fifty-three Member States, MERCOSUR for its five Member States and several Associated States, or ASEAN for its ten Member States. These steps could include a collective remedy for failure to provide information or data they may require from manufacturers or countries of manufacture, as discussed above. These steps could also include enforcing a decision by a Member State to refuse entry of a particular drug from a particular manufacturer in a particular country to apply across all member states of a regional body. Further, a regional body could take steps to prohibit the import of medicines from a particular manufacturer or a particular country if products from a particular manufacturer or country are consistently shown to be substandard.

Putting this type of authority into the hands of importing countries and regional organizations would give greater political and economic weight when engaging with companies and countries to change how they address quality and safety in the e manufacturing and distribution of medicines. Such collective action ensures that countries and people that are most affected by the trade in substandard medicines have an effective way to address the problem. Taking such collective action by affected states does not run afoul of agreements administered by the World Trade Organization (WTO) or regional or bilateral trade agreements. Indeed, early in the COVID-19 pandemic, the Secretariat of the WTO issued a document on this point, stating that:

illicit trade also poses a considerable challenge to government efforts to ensure product quality and safety. The COVID-19 pandemic forced governments to develop new solutions to ensure regulations facilitate access to essential health products. To meet surging demand for products like personal protective equipment (PPE), governments introduced a range of emergency regulatory measures to accelerate approval and access to medical goods during the COVID-19 pandemic. These included streamlining conformity assessment procedures (CAPs) (e.g., emergency use authorization pathways which reduced the level of controls applied by governments), simplifying product labelling, or deepening reliance on decisions of other regulators [30].

Countries should take advantage of the newly launched TRVST System as a complement to other actions that may be taken as outlined above. The TRVST System will enable a country to quickly locate medicines from particular manufacturers that have been identified as sub-standard and remove them from the market.

The CoPP System is an important tool to enable safe international trade in medicinal products, but the CoPP System has been used in a manner that limits its desired result. Achieving the stated objectives of the CoPP System requires a higher level of transparency and greater attention to compliance than has been the case to date. This article recommends measures to improve the use of and results from the Coppa system. Given that it has been a decade since NASEM’s pathbreaking report, it is time to convene experts, regulators, and researchers through a similar forum to not only explore how these measures may be efficiently implemented but to systematically analyze the actors, gaps, and remedies that can avoid unnecessary illness and death resulting from medicines that are supposed to cure and heal.
